# A multimedia intervention to enhance recruitment to clinical trials in primary care and community settings: process of development and evaluation

**DOI:** 10.1186/1745-6215-14-S1-P90

**Published:** 2013-11-29

**Authors:** Peter Bower, David Collier, Sandra Eldridge, Jonathan Graffy, Anne Kennedy, Peter Knapp, Adwoa Hughes-Morley, Jo Rick, Chris Salisbury, Nicola Small, David Torgerson, Shaun Treweek, Peter Wallace

**Affiliations:** 1 University of Manchester, Manchester, UK; 2 Barts and The London, London, UK; 3 Queen Mary University of London, London, UK; 4 University of Cambridge, Cambridge, UK; 5 University of Southampton, Southampton, UK; 6 University of York, York, UK; 7 University of Bristol, Britsol, UK; 8 University of Dundee, Dundee, UK; 9 University College London and NIHR PCRN, London, UK

## Background

The aim of the MRC START programme is to improve the evidence-base concerning recruitment to trials by nesting innovative recruitment interventions across multiple host trials. This poster presentation will outline the development of a web-based multimedia intervention to be tested in the MRC START programme.

## Methods

Intervention content was informed by four elements: (a)core themes were generated by team members, (b) a review of factors identified by patients as being determinants of decisions about trial participation; (c) input from members of a patient and public involvement (PPI) forum; (d) input from qualitative experts on patient health experiences (healthtalkonline.org).

## Results

Nineteen reviews were included. Multimedia interventions offer a platform for learning which can include *study-specific information* (e.g. study purpose, risks), and *generic information* (e.g. confidentiality). There was evidence that multimedia interventions improved various outcomes including perceived risk, personal choice and feeling informed.

PPI forum members and qualitative experts developed study-specific components involving bespoke themes such as investigator details and benefits of participation, and generic information components including informed consent, randomisation and confidentiality. Existing video clips of patients discussing their experiences of participation were carefully matched to these components.

## Delivery

The multimedia intervention was developed by a commercial company for use on a range of platforms including desktops and smartphones. The MRC START programme is testing the effects of the intervention on recruitment rates, conducting nested trials of the intervention among existing primary care and community trials. Example websites will be on display with the poster.

